# 

**Published:** 1895-11-09

**Authors:** 


**Notices of Births, Deaths, and Marriages** are inserted in this column at a charge of 2s. 6d. for an announcement not exceeding four lines.

**Notice to Advertisers and Subscribers.**

All Advertisements, Orders for Copies of THE HOSPITAL and Business Communications should be addressed to **THE MANAGER, (Not to The Editor)**, THE HOSPITAL, 428, Strand, London, W.C.

The Cost of Subscription, post free, is as follows:—Per week, 2½d.; per quarter, 2s. 8d.; per half-year, 5s. 3d.; per annum, 10s. 6d. Foreign:—Per Annum, 15s. 2d.; payable, in all cases, in advance.

### NOTICE TO CORRESPONDENTS.

All MS., Letters, Books for Review, and other matters intended for the Editor should be addressed THE EDITOR, THE LODGE, PORCHESTER SQUARE, LONDON, W.

The Editor cannot undertake to return rejected MS., even when accompanied by stamped directed envelope.

**"Burdett's Hospital Annual," 1895.**

Sixth year of publication. 950 pp. Scarlet cloth, gilt lettered. Price 5s. A most complete guide to British, Colonial, Indian, and American Hospitals, Dispensaries, Nursing and Convalescent Institutions, and Asylums. A few complete sets of the preceding five issues of the "Annual" may still be obtained on application to the Publishers, THE SCIENTIFIC PRESS (Limited), 428, Strand, London, W.C.

**NOTICE.—Vol. XVIII. of "The Hospital" (April to September, 1895), in handsome cloth binding, is now on sale at the Office of this Journal. Price 6s. Cases for binding the Numbers contained in this Volume, 1s. 6d. Index to Vol. XVIII., 2½d., post free.**

**The Monthly Part for November is now ready, price 6d.; by post, 8d.**

### SCRAPS AND GLEANINGS.

The foundation stone of the Cottage Hospital at Accrington has just been laid.

One of the most prominent medical women of Buffalo has just lost her life in a railway accident.

Miss Harriet Hopkins, of New Steyne, Brighton, has bequeathed £1,000 to the Throat Hospital, Golden Square.

A dance and concert were given recently by the Park, the Queen, and the Botanic Wards of the Hull Royal Infirmary in aid of the funds of the institution.

The annual dinner of past and present students of the Charing Cross Medical School has been held at the Holborn Restaurant. Mr. C. J. Worlett, F.R.C.S., was in the chair.

The anniversary service of the Westminster Hospital was held last week in the hospital chapel, and an organ recital was given by Professor Bridge, organist to Westminster Abbey.

The name of "Pasteur" is to be given henceforth to the Municipal College at Arbois, Pasteur's birth-place; and a statue is shortly to be erected in the town in commemoration of that fact.

The practice of homœopathy has been a difficult matter to bring about in Peru; an American physician has been struggling to obtain permission to do so, and has only succeeded in his endeavour after five years' effort.

The church parade of Friendly Societies in aid of the funds of the Devizes Cottage Hospital took place quite recently, through which effort a substantial sum was added to the funds of that deserving institution.

The Queen of Saxony goes to the hospitals two or three times a week. The beautiful hospital just outside Dresden is called after her, "Carola House." The Band of Nursing Sisters working in the town was formed by her.

At the end of last week an important conference of representatives of the various local authorities in the County of Staffordshire was held at Stafford for the purpose of considering a proposed isolation hospital for the county.

A curious fact was lately discovered about the skull of a man who had died from delirium tremens. The skull when opened was found to contain alcoholic vapour; this was permitted to escape, and when ignited burnt with a bluish flame.

We learn that a lady has been unanimously elected vaccination officer by the Houghton-le-Spring Board of Guardians. The present officer of this department is Mrs. Emily Stokoe, who has succeeded in the place of her father, recently deceased.

The fourteenth annual bulletin of the French Cremation Society states that in Paris alone there have been more than 20,000 bodies burned since the beginning of the movement. The French Society counts amongst its members a large proportion of women.

The finances of the Guest Hospital, Dudley, have lately been well supported by the working classes of the district; out of the annual income for 1894 of £1,500 from voluntary contributions, nearly half of this sum has been supplied by working men.

The veterinary societies of Melun are combining with the agricultural of the same locality to raise a subscription list with the view to erecting a statue to Pasteur. It was at Melun where the famous French scientist effected a series of his most notable vaccine experiments.

A salutary recommendation has been presented to the members of the Municipal Council of Paris that, before children recovering from diphtheria be permitted readmittance to schools, they shall be obliged to obtain a certificate declaring their freedom from contagion of the disease.

A sale of work in aid of the Westminster Refuge, Great College Street, and St. Helena's Hospital Home, will be held in the Jerusalem Chamber, Westminster Abbey, next week, Princess Christian having promised to open the proceedings on Tuesday, Nov. 12th, at twelve o'clock.

Dr. Behring's comparison of the effects of the anti-toxin treatment in Berlin Hospitals is an interesting one. In the two hospitals thus compared, the one employing the special treatment, the other not, the deaths showed 17.7 per cent. as against 45 per cent. where the anti-toxin was excluded.

The educational value of the Royal Orthopædic and Spinal Hospital at Birmingham continues to be much appreciated. A number of probation nurses have passed through a term of training, and students of the Birmingham Medical School have diligently followed the surgical practice and clinical teaching.

The west wing of the new children's hospital at Edinburgh has been named after Lady Caroline Charteris. Lady Jane Duncan, who has already contributed £11,500 to the funds, has intimated her intention of giving a further donation of £7,000 to endow the 24 cots in memory of her sister, Lady Caroline Charteris.

At the 113th anniversary of the Nottingham General Hospital the collection made after the special service at the cathedral realised £222. Allusion was again made at the general meeting of the hospital to the yearly increasing demands upon the institution, and the inadequacy of the present accommodation to meet the demands of a growing population.

The Middlesex Hospital has quite recently added some portable Merryweather hand fire-pumps to their other precautionary arrangements for dealing with fire. These hand fire-pumps are especially useful in outbreaks of fire which are not of a really alarming nature, as they do not cause the damage by water which frequently follows the action of the hydrants.

A special meeting has been held at the Brighton Pavilion with the object of obtaining additional support to the funds of the Sussex County Hospital, the subscription list of which institution shows a serious falling off. In fact, since 1891, the subscriptions have shown a "progressive decrease" to the extent of £500; and the committee have had to draw upon legacies to discount current expenses.

A great bazaar is to be held at Cardiff next year in aid of the funds of the Cardiff Infirmary; money is very seriously needed to meet the outlay of the extension work. This cost the committee more than £14,000, only half of which sum has been met. The new wards cannot be opened for lack of funds, and the bazaar scheme, inaugurated by Lord and Lady Windsor, has met with a hearty response from the surrounding neighbourhood.

### BOOKS RECEIVED.

J. P. LEGG AND CO.

"The Diseases of Children's Teeth, their Prevention and Treatment." By R Denison Pedley.

SWAN SONNENSCHEIN AND CO.

"A Woman's Word to Women." By Mary Scharli b, M.D.

"Stories for Ten-Year-Olds." By Frances Wace Saunders.

LEADENHALL PRESS.

"Master Jimmy's Fables." By St. John Browne. Illustrated by Walter Warren.
"The Cavendish Lecture on Dreamy Mental States." By Sir James Crichton-Browne, M.D.

S. C. AND E. C. JACK.

"The Art of Laundry-work." By Florence B. Jack.

ISBISTER AND CO. (LIMITED).

"Women in the Mission Field." By Rev. Augustus R. Buckland, M A.

BALLIERE, TINDALL, AND COX.

"The Tallerman-Sheffield Patent Localised Hot-air Bath."
"Aids to the Analysis of Foods and Drugs." By S. H. Pearmain and C. G. Moor.

WILLIAM HEINEMANN.

"Movement." By E. J. Marly. Translated by Eric Pritchard, M.A.

RICHARD BENTLEY AND SON.

"A Life's History." By J. Reddie Mallett.

S. W. PARTRIDGE AND CO.

"The Zenana." Vol. II.

COUNTY COUNCIL OF DUMBARTON.

"Report on Pollution of the Gareloch." By Mr. Francis C. Buchanan and John C. M'Vail, M.D.

BLACKIE AND SON (LIMITED).

"Elementary Physiology." By J. R. Ainsworth Davis, B.A.
"A Manual of Physiology." (University Series.) By G. N. Stewart, M.A.

### THE STUDENT OF MEDICINE.

### APPOINTMENTS.

C. E. G. Bateman, L.R.C.P., L.R.C.S.Edin., appointed Medical Officer for the Workhouse and Walsingham District of the Walsingham Union. E. Kennedy Birley, M.R.C.S., L.R.C.P. Lond., appointed House Surgeon to the Chorley Dispensary and Cottage Hospital. A. Boulton, M.R.C.S.Eng., appointed Medical Officer of Health to the Horncastle Rural Sanitary District. W. J. Carmichael, M.B., C.M.Aberd., appointed Surgeon and agent to the Coastguard at Collieston, and Medical Officer for the Parish of Slains. N. F. Edwards, M.B., Ch. B. Vict., appointed House Physician to the Swansea Hospital. A. H. Evans, appointed Resident Obstetric Assistant to the Westminster Hospital. Dr. G. E. Fleming, appointed Medical Officer for the Downham District of the Ely Union. T. W. H. Garstang, M.R.C.S., Medical Officer of Health to the Knutsford Urban District Council, appointed Medical Officer of Health to the Bucklow Rural District Council, late Altrincham Rural Sanitary Authority. F. R. Gibbs, M.R.C.S., L.R.C.P.Lond., appointed Medical Officer for the Eighth District of the Wycombe Union. W. Gordon, M.A., M.D., B.C.Cantab., M.R.C.P., appointed Physician to the West of England Eye Infirmary, Exeter. J. Hardie, M.B., F.R.C.S.E, appointed Examiner in Anatomy at the Royal College of Surgeons, Edinburgh. R. W. Jolly, M.R.C.S.Eng., appointed Medical Officer of Health for Peterborough. H. W. Morley, M.R.C.S., L.R.C.P., formerly Assistant House Surgeon, appointed House Surgeon to the Royal Portsmouth Hospital. C. Pollard, M.D., F.R.C.S., appointed Medical Officer for the Holt District of the Martley Union. R. M. Henry Randell, M.D.Lond., M.R.C.S.Eng., appointed Honorary Medical Officer to the Beckenham Cottage Hospital. F. J. Stevens, B.A.Oxon., M.R.C.S., D.P.H., appointed Medical Officer of Health to the Camberwell Sanitary District. T. M. Tilbetts, M.B.Lond., M.R.C.S.Eng., L.R.C.P.Lond., appointed Medical Officer of Health for the Quarry Bank Urban Sanitary District. Waldemar S. West, M.A., M.B., B.C.Cantab, appointed Resident Medical Officer to the Royal Hospital for Diseases of the Chest, City Road, E.C. General Infirmary, Leeds: E. S. Steward, M.R.C.S., L.R.C.P., appointed Resident Ophthalmic Officer; J. Nicholson, M.R.C.S., L.R.C.P., appointed Resident Medical Officer at Ida Hospital; C. E. Ligertwood, M.B., B.Ch.Vict., appointed House Physician; R. H. Trotter, M.B., B.Ch.Vict., appointed House Surgeon; A. McKenzie, B.A.Cantab., M.R.C.S., L.R.C.P., appointed House Surgeon; A. Tait, M.B. Lond., appointed Resident Obstetric Officer; and W. A. Stott, re-appointed Pathological Curator.

### VACANCIES.

### Resident Medical Officers.

Royal Portsmouth Hospital.—Assistant House Surgeon; appointment for six months. Honorarium of £15 15s., and board and residence, and is renewable for a further period of six months. Applications and testimonials to J. A. Byerley, Secretary, before November 14th

Denbighshire Infirmary, Denbigh.—House Surgeon; must be duly qualified to practise medicine and surgery, and be conversant with the Welsh language. Salary, £80 per annum, with board, residence, and washing. Applications and testimonials to W. Vaughan Jones, Secretary, by December 2nd.

Tiverton Infirmary and Dispensary.—House Surgeon and Dispenser; registered, unmarried. Salary £105, with lodgings, attendance, fire, and lights. Must serve two years, if required. No canvassing allowed. Applications, with testimonials, to Arthur Fisher, hon. sec., Tiverton, Devon, by November 25th inst.

Whitehaven and West Cumberland Infirmary.—House Surgeon; doubly qualified. Salary £120 per annum, and £30 per annum for dispensing, with furnished apartments and attendance. Applications and testimonials to Tyson Kitchen, Secretary, by November 9th.

### Non-Resident Medical Officers.

Brown Animal Sanatory Institution.—Professor Superintendent. Salary £250 per annum. Applications to the Registrar of the University of London, Burlington Gardens, W., by November 15th.

### UNIVERSITIES AND COLLEGES.

### Examining Board in England by the Royal Colleges of Physicians and Surgeons.

The following gentlemen passed the second examination of the Board in the subjects indicated.

Wednesday, October 9th.

ANATOMY AND PHYSIOLOGY.—L. O. Fuller, E. A. Bullmore, W. S. Sheldon, and A. E. Francis, students of University College, London; N. J. Roche and V. S. Partridge, of Charing Cross Hospital; C. L. Francia, B. E. G. Bailey, E. N. Berryman, A. H. Brewer, and J. K. S. Fleming, of St. Bartholomew's Hospital; W. R. Cazenove, H. P. W. Barrow, J. Howells, and N. Williams, of Guy's Hospital; F. Bawtree, of St. Thomas's Hospital; W. Meade, of St. George's Hospital; H. Hipwell, of Middlesex Hospital; K. de R. Bell and H. N. Harness, of King's College, London.

ANATOMY ONLY.—H. P. Shanks, of University College, London; and E. C. Plummer, of King's College, London.

Sixteen gentlemen were referred in both subjects, and one in Anatomy only.

Thursday, October 10th.

ANATOMY AND PHYSIOLOGY.—G. G. Campbell, E. P. Court, W. H. Carzaly, J. Dalebrook, and N. H. Joy, of St. Bartholomew's Hospital; P. R. Tarbet and D. L. K. Pritchard, of University College, London; A. D. Lewis, H. W. Fox, and W. W. Harrison, of Guy's Hospital; H. C. Adams, of Middlesex Hospital; J. C. S. Oxley, of St. Thomas's Hospital; J. I. W. Morris and L. Lindop, of St. Mary's Hospital; R. B. Ainsworth and F. B. Thompson, of St. George's Hospital; F. J. A. Beringer, of London Hospital.

ANATOMY ONLY.—A. W. Penrose, of Guy's Hospital; A C. Bird, of St. Thomas's Hospital and Mr. Cooke's School of Anatomy and Physiology; W. Amsden, of St. Bartholomew's Hospital and Mr. Cooke's School of Anatomy and Physiology; F. H. Lawson and E. S. Shave, of Middlesex Hospital; E. J. Distin and J. J. C. Hamilton, of King's College, London.

PHYSIOLOGY ONLY.—G. H. Dominy, of St. Thomas's Hospital; and C. S. Agnew, of Charing Cross Hospital.

Twelve gentlemen were referred in both subjects one, in Anatomy only, and one in Physiology only.

### From A. and C. BLACK'S LIST.

**Text-Book of Operative Surgery.** By Dr. TH. KOCHER, Professor of Surgery and Director of the Surgical Clinic in the University of Bern. Translated by special permission of the Author from the Second Enlarged and Improved German Edition, by HAROLD J. STILES, M.B., C.M., Senior Demonstrator of Surgery in the University of Edinburgh; Assistant Surgeon, Royal Edinburgh Asylum for Sick Children. Illustrated with 185 cuts in text. Demy 8vo., cloth, price 20s.

**The Senile Heart.** Its Symptoms, Sequelæ, and Treatment. By GEO. W. BALFOUR, M.D., LL.D., Consulting Physician to the Royal Infirmary and to the Royal Public Dispensary, Edinburgh. Crown 8vo., cloth, price 5s.

**Plea for a Simpler Life.** By GEORGE S. KEITH, M.D., F.R.C.P.E. Crown 8vo., cloth, price 2s. 6d.

**Text-Book of General Pathology and Pathological Anatomy.** By Professor RICHARD THOMA, of Dorpat. Translated by ALEX. BRUCE, M.D., Lecturer on Pathology, Surgeons' Hall, Edinburgh. [*Vol. I. in preparation.*

A. AND C. BLACK, Soho Square, London, W.

Just Published. Crown 8vo., 6s.

### PIONEER WORK IN OPENING THE MEDICAL PROFESSION TO WOMEN:

**Autobiographical Sketches.**

BY DR. ELIZABETH BLACKWELL.

LONDON: LONGMANS, GREEN, AND CO.

### PAMPHLETS ON NURSING.

No. 2, Now Ready, price 1½d. per copy, or 1s. per dozen.

**A GREAT MOVEMENT—THE NURSES' CO-OPERATION.**

By Mrs. FURLEY SMITH.

Also No. 1, price 1½d. per copy, or 1s. per dozen.

**THE ADVANTAGES AND PRIVILEGES OF A TRAINED DISTRICT NURSE, AND THE DUTIES OF THE DISTRICT TOWARDS HER.**

By HENRY C. BURDETT.

London: THE SCIENTIFIC PRESS (Limited), 428, Strand, W.C.

*In the Press. Price 5s.*

### BURDETT'S OFFICIAL NURSING DIRECTORY

A DIRECTORY, NOT A REGISTER.

The Hospital states: "It is well to bear in mind the difference and distinction which exists between a register and a directory. A register implies rights, and suggests that those who are not upon it are in some way or another inferior beings. But a directory is another matter. A directory gives no rights; insertion in it implies no adherence to any particular party or any particular opinion, and in no way interferes with entry on the register of any association, or the list of any institute, to which the individual may belong. All that a directory professes to do is to give information. The 'Medical Register' is not a mere list of qualified medical men; it is admission to the Register, which itself makes him qualified. For years, perhaps even now, 'Churchill's Medical Directory' contained the names of medical men who, so far as public appointments were concerned, were unqualified; it also contains the names of homœopathists, whom four out of every five medical men will not meet; and it certainly contains the names of many for whose moral character we should not like to vouch. But it gives full and accurate information about them all, and for the sake of that information it is constantly consulted both by the public and the doctors."

**The above work may be subscribed for at a reduced price by those whose names appear in the book, and who supply full particulars on a form which can be obtained upon application to the Publishers,**

THE SCIENTIFIC PRESS (Lim.), 428, Strand, London, W.C.

### NURSING BOOKS AT FULL DISCOUNT.

*LIST POST FREE.*

The Theory and Practice of Nursing (Percy Lewis, M.D.), 3/6 for **2/8.**

The Nurse's Dictionary (Honnor Morten), 2/- for **1/6.**

The Nurse's Case Book, 6d. for **3½d.**

### THE SCIENTIFIC PRESS LIST.

Now Ready. With numerous Illustrations, demy 8vo., blue buckram, gilt, 3s. 6d.

### DIET IN SICKNESS AND IN HEALTH.

By Mrs. ERNEST HART,

*Formerly Student of the Faculty of Medicine of Paris, and of the London School of Medicine for Women.*

With an Introduction by Sir HENRY THOMPSON, F.R.C.S., M.B. Lond.

A Practical Treatise on Diet both in Sickness and Health, based upon the most recent scientific and medical knowledge, accompanied by a Series of Valuable Recipes for the Feeding of Invalids and others.

Sir Henry Thompson, in his Introduction, says :—"I do not hesitate to express my opinion that the present volum, forms a handbook to the subject, which will not only inter est the dietetic student, but offer him, within its modest compass more complete epitome thereof than any work which has yet come under my notice."

Third and Revised Edition. Demy 8vo., 209 pp., profusely Illustrated with Copyright Portraits and Drawings. Price 2s. 6d.

**HOW TO BECOME A NURSE**: And How to Succeed. A complete guide to the Nursing Profession for those who wish to become Nurses, and a useful book of Reference for Nurses who have completed their training and seek employment. Compiled by HONNOR MORTEN (Author of "Sketches of Hospital Life," "The Nurse's Dictionary," &c.)

"To those who are frequently appealed to by girls or young women, as to the steps they should take to become nurses, this book of Miss Morten's must prove a perfect godsend."—*British Medical Journal.*

Second and Revised Edition. Demy 16mo., suitable for the apron pocket, handsomely bound in cloth, 140 pp. Price 2s.; in handsome leather, gilt, price 2s. 6d. net.

**THE NURSES' DICTIONARY OF MEDICAL TERMS AND NURSING TREATMENT.**—Compiled for the use of Nurses by HONNOR MORTEN. Containing descriptions of the principal Medical and Nursing Terms and Abbreviations, Instruments, Drugs, Diseases, Accidents, Treatments, Physiological Names, Operations, Foods, Appliances, &c., &c., encountered in the Ward or Sick-room.

"Should be at the disposal of every nurse."—*Birmingham Medical Review.*

Second Edition. Enlarged, demy 8vo., profusely illustrated, nearly 200 pp., cloth. Important Text-book for Asylum Attendants. Price 2s. 6d.

**MENTAL NURSING.** A text-book specially designed for the instruction of Attendants on the Insane. By WILLIAM HARDING, M.B. The want of a complete book for the instruction of asylum attendants has been long felt, and it is with confidence that the publishers recommend this work to the managers of all Institutions for the Insane.

200 pp., Crown 8vo., cloth, 3s. 6d. Profusely Illustrated with Original Drawings, with list of Instruments required, and a Glossary of Terms.

**OPHTHALMIC NURSING.** By SYDNEY STEPHENSON, M.B., F.R.C.S.E., Surgeon to the Ophthalmic School, Hanwell, W., &c., &c. This is a valuable contribution to Nursing literature on a subject hitherto never handled with special reference to Nursing. On account of its compact and clear arrangement, and its numerous illustrations, it is specially recommended to the use of the Nursing Profession.

"May very advantageously be studied by nurses and clinical clerks, or dressers who are about to enter the Ophthalmic service of a hospital."—*The Lancet.*

Crown 8vo., imitation cloth, 5s. With 10 plates and many Illustrations in the Text, and Fac-simile Autograph Letters from the late Sir Andrew Clark, M. Pasteur, &c.

**INFANT FEEDING BY ARTIFICIAL MEANS**: A Scientific and Practical Treatise on the Dietetics of Infancy. By S. H. SADLER, Author of "Suggestions to Mothers," "Management of Children," "Education."

Crown 8vo., cloth gilt, price 2s. 6d.

**THE MOTHER'S HELP AND GUIDE TO THE DOMESTIC MANAGEMENT OF CHILDREN.** By P. MURRAY BRAIDWOOD, M.D., formerly Senior Medical Officer to the Wirral Hospital for Sick Children.

Demy 8vo., cloth gilt, 260 pp. Price 3s. 6d.

**ART OF FEEDING THE INVALID.** By a MEDICAL PRACTITIONER and a LADY PROFESSOR OF COOKERY. A popular treatise on the most frequent disorders; with detailed lists of suitable diet and original recipes for daily use. This important work has been undertaken to supply a want long felt by Matrons and Lady Superintendents in Hospitals and Institutions of all kinds where invalids are treated, as well as in private households. It is of the greatest value to nurses in private practice.

Demy 8vo., 200 pp., cloth boards, copiously Illustrated, price 3s. 6d.

**SURGICAL WARD-WORK AND NURSING.** By ALEXANDER MILES, M.D. (Edin.), C.M., F.R.C.S.E. A practical manual of clinical instruction for Nurses and Students in the Wards. Concisely though comprehensively treated.

Crown 8vo., 500 pp., Second Edition, 7 Plates, 18 Illustrations, Charts, &c., price 7s. 6d. net.

**NURSING.** By ISABEL ADAMS HAMPTON.

"It is not too much to say that in no single volume yet published has the subject been so scientifically, carefully, and completely treated as it has been by Miss Hampton. . . . Whether as a teaching manual or as a book of reference, the thoroughly practical instruction which it conveys is sure to obtain a wide circulation."—*The Hospital.*

Demy 8vo., cloth extra, price 3s. 6d. (post free). Popular edition, stitched, price 1s. 6d.

**OUTLINES OF INSANITY.** A Popular Treatise on the Salient Features of Insanity. By FRANCIS H. WALMSLEY, M.D., Medical Superintendent of the Darenth Asylum, Metropolitan Asylums Board, Member of Council of Medico-Psychological Association.

A clearly written manual in which the various features of mental disorder are fully expounded. The author's style is interesting and the reader's attention is riveted throughout.

Crown 8vo., cloth gilt, Illustrated, price 5s.

**HELPS IN SICKNESS AND TO HEALTH**: Where to Go and What to Do. Being a Guide to Home Nursing and a Handbook to Health in the Habitation, the Nursery, the Schoolroom, and the Person, with a chapter on Pleasure and Health Resorts. By HENRY C. BURDETT, Author of "Hospitals and Asylums of the World," "Pay Hospitals of the World," "Cottage Hospitals, General, Fever, and Convalescent," &c., &c.

"It would be difficult to find one which should be more welcome in a household than this unpretending but most useful book."—*The Times.*

In the Press. Ready immediately. Greatly Enlarged and entirely Revised Edition. Profusely Illustrated. Crown 8vo, c'oth. Price 3s. 6d.

**NURSING: ITS THEORY AND PRACTICE.** By PERCY G. LEWIS, M.D., M.R.C.S. Being a complete Text-book of Medical, Surgical, and Monthly Nursing.

This new edition is enlarged by over 50 pp., with special new chapters on monthly nursing and confinements.

LONDON: THE SCIENTIFIC PRESS (LIMITED), 428, STRAND, W.C.

